# Decontamination of 16S rRNA gene amplicon sequence datasets based on bacterial load assessment by qPCR

**DOI:** 10.1186/s12866-016-0689-4

**Published:** 2016-04-23

**Authors:** Vladimir Lazarevic, Nadia Gaïa, Myriam Girard, Jacques Schrenzel

**Affiliations:** Genomic Research Laboratory, Department of Genetics and Laboratory Medicine, Department of Medical Specialties, Geneva University Hospitals, Rue Gabrielle-Perret-Gentil 4, CH-1211, Geneva, 14 Switzerland

**Keywords:** Contaminant DNA, Bacterial communities, 16S rRNA gene sequencing, Microbiome

## Abstract

**Background:**

Identification of unexpected taxa in 16S rRNA surveys of low-density microbiota, diluted mock communities and cultures demonstrated that a variable fraction of sequence reads originated from exogenous DNA. The sources of these contaminants are reagents used in DNA extraction, PCR, and next-generation sequencing library preparation, and human (skin, oral and respiratory) microbiota from the investigators.

**Results:**

For *in silico* removal of reagent contaminants, a pipeline was used which combines the relative abundance of operational taxonomic units (OTUs) in V3–4 16S rRNA gene amplicon datasets with bacterial DNA quantification based on qPCR targeting of the V3 segment of the 16S rRNA gene. Serially diluted cultures of *Escherichia coli* and *Staphylococcus aureus* were used for 16S rDNA profiling, and DNA from each of these species was used as a qPCR standard. OTUs assigned to *Escherichia* or *Staphylococcus* were virtually unaffected by the decontamination procedure, whereas OTUs from *Pseudomonas*, which is a major reagent contaminant, were completely or nearly completely removed. The decontamination procedure also attenuated the trend of increase in OTU richness in serially diluted cultures.

**Conclusions:**

Removal of contaminant sequences derived from reagents based on use of qPCR data may improve taxonomic representation in samples with low DNA concentration. Using the described pipeline, OTUs derived from cross-contamination of negative extraction controls were not recognized as contaminants and not removed from the sample dataset.

**Electronic supplementary material:**

The online version of this article (doi:10.1186/s12866-016-0689-4) contains supplementary material, which is available to authorized users.

## Background

The development of PCR and next-generation sequencing techniques has facilitated studying microbial communities without it being necessary to culture individual members. Because growth requirements vary greatly among different species, and for some species growth conditions have not yet been determined, molecular methods in microbiota investigations are advantageous. However, culture-free approaches may introduce biases in the experimental pipeline, starting from DNA extraction through the generation of sequencing libraries to data analysis. Identification of unexpected taxa in datasets derived from low-density microbiota [1–3], diluted mock communities [4], and cultures [5], demonstrated that a variable fraction of sequence reads originated from exogenous DNA. The sources of these contaminants are reagents used in DNA extraction, PCR, and next-generation sequencing library preparation, and possibly human (skin, oral, and respiratory) microbiota from the investigators [6].

Sample datasets can be decontaminated by removing sequence reads assigned to operational taxonomic units (OTUs) found in negative extraction controls (NECs). Bioinformatics pipelines for performing microbiome analysis such as Qiime [7] facilitate performing this step in an automated manner. Some OTUs identified as contaminants across different studies were repeatedly assigned to the same species or genera [5]. However, an OTU that corresponds to the genuine member of the microbiota of interest may also be found in relevant NECs. It has been suggested not to remove OTUs identified in NECs if they are biologically expected in the given sample type [5]. The distinction between expected and unexpected OTUs in a given sample type may not be always straightforward. For example, *Propionibacterium* is a known reagent contaminant but it is genuinely present in the skin microbiota in proportions that vary between individuals [3]. Similarly, *Stenotrophomonas*, another common reagent contaminant, emerged as a new airway pathogen [4, 8], that may complicate the analysis of respiratory tract samples.

The need to recognize as many contaminants as possible based on differences in the relative abundance of bacterial taxa between NECs, low-density samples, and high-density samples has been highlighted [5, 9]. Others proposed that contaminant OTUs excluded should be those whose relative abundance in NECs is above a given threshold [10]. Inverse correlation of a taxon relative abundance with bacterial load as an indicator of a possible reagent contaminant was initially described for bacterial genera in a mock community [4] and subsequently confirmed at the OTU level in cultures and ‘real’ microbiota samples [2, 3, 5, 11].

The removal of OTUs whose mean relative abundance in NECs is higher than that of microbiota samples of interest has been used to decontaminate datasets obtained by 16S rRNA gene amplicon sequencing of relatively low-density skin and respiratory tract bacterial communities [2, 3]. However, the absolute abundance of certain OTUs may be substantially higher in microbiota samples than in relevant NECs, even if their relative abundance shows the opposite pattern. It is advisable not to remove such OTUs, as they correspond to the microbiota of interest. Here, we further develop this approach by combining relative abundance of OTUs with bacterial load in DNA extracts assessed by quantitative real-time PCR (qPCR).

## Results

### Bacterial load determined by culture and qPCR

**Fig. 1:**
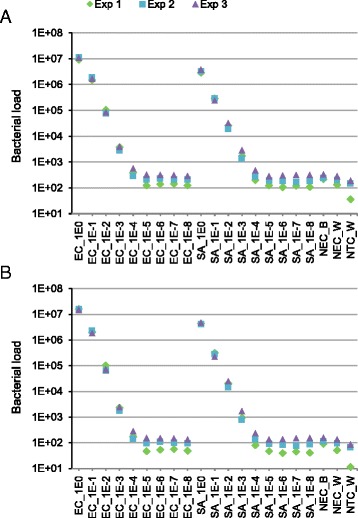
Bacterial load assessed by qPCR. The universal bacterial primers used in qPCR target the V3 segment of the 16S rRNA gene. Bacterial loads were determined using the standard curves obtained with *S. aureus* MW2 (**a**) or *E. coli* DH5α (**b**) genomic DNA. The *S. aureus* and *E. coli* genomes weigh approximately 2.9 and 4.8 fg and contain six and seven 16S rRNA gene copies, respectively. Each symbol (Exp1, Exp2 and Exp3) corresponds to the series of aliquots processed at a given point and represents the mean of duplicate measurements with relative deviations from the mean <2.5 %. Bacterial load is expressed as the number of *E. coli* or *S. aureus* genome equivalents in 1 μl of DNA extract. Serial decimal dilutions of the master stock are indicated from 1E0 (no dilution) to 1E-8 (10^−8^). SA, *S. aureus*; EC, *E. coli*. NEC_B, negative extraction controls obtained by substituting culture for lysis buffer; NEC_W, negative extraction controls obtained by substituting culture for water; NTC_W, no template (qPCR) control reactions performed by substituting DNA extract for water

*Staphylococcus aureus* and *Escherichia coli* overnight cultures were washed and concentrated, and resulted in 3.5x10^10^ and 5.3x10^9^ colony-forming units (CFU)/ml, respectively. Serial decimal dilutions of these master stocks were aliquoted in triplicate and frozen. DNA was extracted from each of the three identical series of aliquots on separate occasions. Serial culture dilutions down to 10^−5^ correlated with decreasing DNA yields in purified extracts determined by qPCR targeting the V3 segment of the bacterial 16S rRNA gene with universal bacterial primers (Fig. 1). Further dilutions (10^−6^–10^−8^) of the master stocks had DNA quantity estimates similar to those of NECs obtained by substituting culture for water (NEC_W) or lysis buffer (NEC_B) in DNA extraction. The lowest DNA concentration was found for no-template controls (NTC_W) in which water was used instead of DNA extract. Bacterial loads determined by qPCR based on *S. aureus* (Fig. 1a) or *E. coli* (Fig. 1b) reference curves showed similar patterns.

### Taxonomic analysis of samples and negative controls

The sequence dataset generated by Illumina sequencing of V3–4 16S rRNA gene amplicons was represented by 9042–176,345 reads per sample after quality filtering and OTU mapping. The proportion of quality-filtered sequences with no hits (with ≥97 % identity) in the Greengenes reference database [12] was 0.63 ± 0.04 % (mean ± SD) for master culture stocks and 4.2 ± 1.9 % for negative controls (NEC_W, NEC_B, and NTC_W). The RDP classifier [13] (with ≥80 % confidence) assigned these sequences to Pseudomonadales (24.1 %), Parcubacteria (11.7 %), Actinomycetales (8.4 %), unclassified Bacteria (14.6 %) and unclassified organisms (7.8 %).

**Fig. 2:**
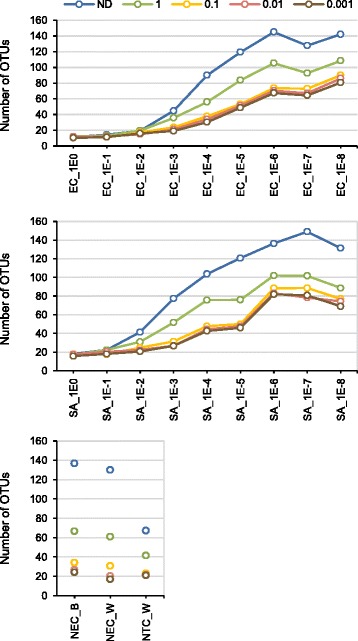
OTU richness across the samples before and after *in silico* decontamination. R-OTU (ratio between mean ‘absolute’ abundance of OTUs in negative extraction controls and culture samples) cut-offs of 1 to 0.001 were applied for decontamination. This ratio was calculated from the relative OTU abundance and qPCR data obtained using the *S. aureus* standard curve. Dilutions of the master stock are indicated from 1E0 (no dilution) to 1E-8 (10^−8^). EC, *E. coli*; SA, *S. aureus*. NEC_W, negative extraction controls obtained by substituting culture for water; NEC_B, negative extraction controls obtained by substituting culture for lysis buffer; NTC_W, no-template PCR controls; ND, no decontamination was performed

A total of 2673 OTUs were identified in the final dataset of which 1718 were found only in samples, 276 were specific to negative controls, and 855 were found in both. OTUs richness in NEC_W and NEC_B were similar to each other but higher than that of NTC_W. In the dataset normalized to the same number of sequences per sample (3500), OTU richness increased as bacterial counts decreased (Fig. 2).

**Fig. 3:**
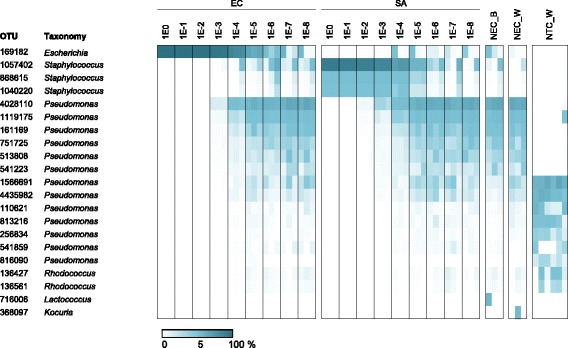
Relative abundance of predominant OTUs. OTUs with a mean relative abundance >1 % in either samples, negative extraction controls or NTC_W are presented. The proportion is indicated by the scale at the bottom of the plot. Dilutions of the master stock are indicated from 1E0 (no dilution) to 1E-8 (10^−8^). For EC, SA, NEC_B and NEC_W, the data obtained from DNA extractions performed on three occasions (Exp1–Exp3) are presented from left to right. NTC_W were performed in duplicate for each of the three series. EC, *E. coli*; SA, *S. aureus*. NEC_W, negative extraction controls obtained substituting culture for water; NEC_B, negative extraction controls obtained by substituting culture for lysis buffer; NTC_W, no-template PCR control

Serial culture dilutions were associated with a decrease in the proportion of sequence reads assigned to *Escherichia* or *Staphylococcus*, and an increase in the relative abundance of reads that derived form contaminants. *Pseudomonas* was major contaminant, and was most abundant in negative controls and highly diluted samples (Fig. 3). Interestingly, OTUs assigned to *Pseudomonas* had different profiles in NECs (NEC_W and NEC_B) and NTC_W. For example, OTU4028110 and OTU1566691, which dominated NECs and NTC_W, respectively, differed in 11 residues in the sequenced V3–4 region. These results show specific contamination of both DNA extraction and PCR reagents. The most abundant OTUs in NTC_W, which corresponded to the contaminants of the PCR reagents, were identified in most NECs, where they were outnumbered by OTUs from contaminants from DNA extraction. However, the OTUs highly abundant in NECs were mostly absent from NTC_W. The similarity of NEC_W to NEC_B and its difference from NTC_W OTU profile indicate that ultrapure water was not the major source of DNA contamination.

In the sequence data from highly diluted *E. coli* cultures, we identified a substantial proportion of OTUs assigned to *Staphylococcus* and, also found *Escherichia* OTUs in the sequence data from diluted *S. aureus*. These results and the fact that *Staphylococcus* has not been previously recognized as reagent contaminant [5] indicate cross-contamination during DNA extraction from samples with high bacterial load, notably by *E. coli* in experiment 1 and by *S. aureus* in experiment 3 (Fig. 3). However, the proportion of cross-contaminants was lower than that of reagent contaminants.

### *In silico* decontamination procedure

**Fig. 4:**
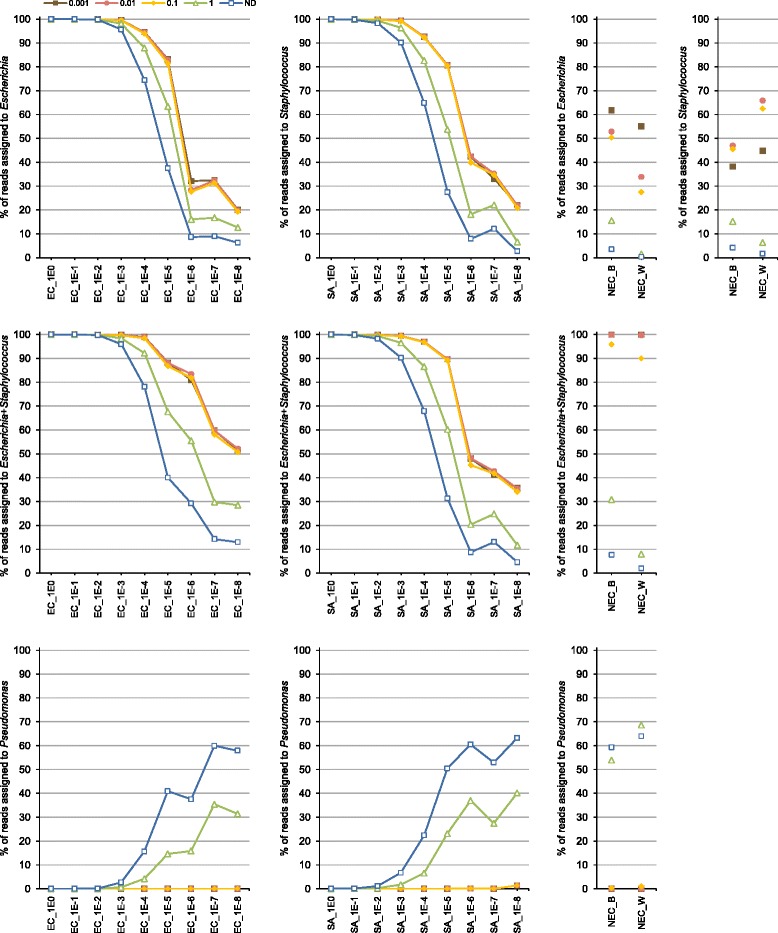
Effect of *in silico* decontamination on taxonomic profiles of culture dilutions and negative extraction controls. Means for three samples obtained in separate DNA extraction experiments are given. The R-OTU (the ratio between mean ‘absolute’ abundance of OTUs in negative extraction controls and culture samples) cut-offs of 1 to 0.001 were applied for decontamination. This ratio was calculated from the relative OTU abundance and qPCR data obtained using the *S. aureus* standard curve. Dilutions of the master stock are indicated from 1E0 (no dilution) to 1E-8 (10^−8^). EC, *E. coli*; SA, *S. aureus*. NEC_W, negative extraction controls obtained by substituting culture for water; NEC_B, negative extraction controls obtained by substituting culture for lysis buffer; ND, no decontamination was performed

To obtain an approximate estimation of the ‘absolute’ abundance of OTUs, expressed in arbitrary units, we multiplied the relative abundance of each OTU by the 16S rRNA gene copy number of a given sample (determined by qPCR). We then calculated the ratio (designated R-OTU) between mean ‘absolute’ abundance of OTUs in NECs and culture samples. The dataset was decontaminated *in silico* using four R-OTU cut-off values (1, 0.1, 0.01 and 0.001), by removing OTUs for which this ratio was exceeded. The proportion of the bacterial genera *Escherichia*, *Staphylococcus* and *Pseudomonas* before and after decontamination are shown in Fig. 4. The decontamination procedure improved the taxonomic profile of low-abundance (diluted) culture samples. For example, at a 10^−5^ dilution, *Staphylococcus* and *Escherichia* corresponded to 27.5 and 37.5 % of reads, respectively, but after decontamination using an R-OTU cut-off of 0.01, they increased to >80 % of reads.

Very few reads (0–0.28 % per sample) assigned to *Escherichia* or *Staphylococcus* were affected by the decontamination procedure using R-OTU of 0.01. *Pseudomonas*, which represented 37–63 % of reads in 10^−5^ to 10^−8^ dilutions, was completely or nearly completely removed by decontamination (except in one *E. coli* 10^−8^ dilution, where it was found at a proportion of 1.4 %). Decontamination of NECs resulted in datasets that contained 99.1–100 % reads assigned to a combination of *Staphylococcus* and *Escherichia* (Fig. 4 and Additional file 1: Figure S1). The profiles of OTU abundance (Fig. 3) point to cross-contamination by both *E. coli* and *S. aureus* during sample handling.

The decontamination procedure also attenuated the trend of increase in OTU richness in serially diluted cultures (Fig. 2).

## Discussion

Our pipeline for removal of reagent contaminants in sequence datasets combines the relative abundance of OTUs in V3–4 16S rRNA gene amplicon datasets with bacterial DNA quantification based on qPCR targeting of the V3 segment of the 16S rRNA gene. Both PCR and qPCR may introduce biases by preferential amplification of certain targets. However, our results showed only a limited impact of organisms chosen to generate the standard curve in qPCR experiment. Decontamination procedures based on *E. coli* and *S. aureus* standard curves resulted in comparable results with R-OTU thresholds of 0.1, 0.01, and 0.001 (Fig. 4 and Additional file 2: Figure S2). These two bacteria are phylogenetically relatively distant, as they belong to two different phyla (*E. coli*, Proteobacteria; *S. aureus*, Firmicutes), have different genome sizes (*E. coli*, 4.7 Mbp; *S. aureus*, 2.8 Mbp) and differ in the number of 16S rRNA gene copy number (*E. coli*, seven; *S. aureus*, six).

The R-OTU cut-offs of 0.1, 0.01 and 0.001 had a similar effect on the removal of contaminants for the two pure bacterial cultures in our study. However, the R-OTU cut-off that best separates contaminants from expected taxa may be influenced by the taxonomic composition and bacterial load of the samples analysed in a given study. Defining the threshold that removes as much contaminant taxa as possible while not affecting the taxa of interest remains an arbitrary choice in both decontamination procedure we used and other approaches.

Although decontamination procedures both *in silico* and in the wet lab are not yet fully developed, they improve the taxonomic profiles of microbiota, thus providing benefits when samples (e.g. clinical specimens) are available in limited amount and/or have low bacterial load. Longer 16S rRNA gene sequences, lower sequencing error rates, and OTU clustering at identity thresholds >97 % may contribute to better distinction between contaminants and ‘real’ OTUs. In addition to further development of bioinformatics and statistical approaches for decontamination after sequencing, it may also be advantageous to reduce DNA contamination of laboratory reagents during their manufacturing and reduce the risk of sample-to-sample contamination during the experiments.

## Conclusions

We show that removal of contaminant OTUs derived from reagents based on the combination of qPCR data and relative abundance of OTUs may improve taxonomic representation in samples with DNA concentrations close to those of NECs. Using the described approach, OTUs derived from cross-contamination, in contrast to those derived from reagents, were not recognized as contaminants and not removed from the dataset. The approach we used in this study may prove useful in situations where OTUs identified in negative controls have higher relative abundance but lower absolute abundance compared with microbiota samples.

## Methods

### Bacterial strains and culture

Fresh colonies of *E. coli* DH5α (Invitrogen, Carlsbad, CA, USA) and *S. aureus* MW2 (strain NRS 123 obtained from the Network of Antibiotic Resistance in *S. aureus* (NARSA)) were inoculated in 20 ml Difco Mueller-Hinton broth medium (BD Diagnostics, Sparks, MD, USA) and incubated overnight with shaking (180 rpm). Three overnight cultures of the same strain were pooled, centrifuged at 1600 *g* for 10 min and washed twice with NaCl 0.9 %. The cells were suspended in 6 ml ddH_2_O (Sigma-Aldrich, Munich, Germany). From each suspension, four serial dilutions 10^−1^ to 10^−8^ were performed by adding 100 μl inoculum to 900 μl ddH_2_O. One series was used immediately for plating onto Mueller-Hinton Agar (BD Diagnostics). CFU were counted after 24-h incubation at 37 °C. The other three series were placed at −20 °C and used for DNA extraction within the following 6 d.

### DNA extraction

DNA was extracted using the NucleoSpin Soil kit (Macherey-Nagel, Düren, Germany). Five hundred microliters of bacterial cell suspensions, 700 μl of lysis buffer SL1, and 100 μl of Enhancer SX were shaken in a NucleoSpin Bead Tube for 4 min at maximum speed on a Vortex-Genie 2 with a horizontal tube holder (Scientific Industries, New York, USA). The lysate was centrifuged at 11,000 *g* for 1 min. Then, we followed the NucleoSpin Soil kit booklet protocol (November 2011/Rev. 03). DNA was eluted in 50 μl of elution buffer SE. Purified DNA was stored at −20 °C.

One *E. coli* and one *S. aureus* dilution series of samples (10^0^–10^−8^) were processed in parallel on three different days. In each batch, an NEC was performed using 500 μl SL1 buffer (NEC_B) or 500 μl ddH_2_O (NEC_W) instead of bacterial suspensions.

### PCR and sequencing

The V3–4 region of the bacterial 16S rRNA genes (*E. coli* positions 341–805) was amplified using template DNA from *E. coli* and *S. aureus* cultures, and from NECs. PCR was performed in a 25 μl volume that contained 5 μl of DNA extract, 12.5 μl KAPA2G Robust HotStart ReadyMix (Kapa Biosystems, Boston, MA, USA), 6.5 ddH_2_O, and 0.5 μl each of 10 μM forward primer 341 F 5’-CCTACGGGNGGCWGCAG-3’ and reverse primer 805R 5’-GACTACHVGGGTATCTAATCC-3’ [14]. Two NTC_W were performed in parallel for each series of bacterial suspensions and NECs using 5 μl ddH_2_O instead of DNA extract. The PCR conditions included an initial denaturation at 95 °C for 3 min, followed by 35 cycles of denaturation at 95 °C for 30 s, annealing at 51 °C for 30 s, and extension at 72 °C for 30 s, with a final extension at 72 °C for 5 min. Each PCR was performed in duplicate and the products were combined. The pooled sample was run on a 2100 Bioanalyzer (Agilent Technologies, Santa Clara, CA) for quality analysis. The primers from the first round of PCR were removed by digesting 5-μl samples with 1 unit Exonuclease I (New England Biolabs, Ipswich, MA, USA) in a total volume of 10 μl Exonuclease I Reaction Buffer (New England Biolabs) at 37 °C for 30 min. The enzyme was inactivated at 95 °C for 15 min. Amplicon barcoding was performed by re-amplification using 1 μl of Exonuclease I-treated first-round PCR, 15 pmol each of forward primer 5’-NNNNNNNNNNTCCTACGGGNGGCWGCAG-3’ and reverse primer 5’- NNNNNNNNNNTGACTACHVGGGTATCTAAKCC-3’ in a 20-μL volume of MyTaq buffer that contained 1.5 units MyTaq DNA polymerase (Bioline, London, UK) and 2 μl of BioStab PCR optimizer (II) (Sigma-Aldrich, Munich, Germany). For each sample, the forward and reverse primers had the same 10-nt barcode sequence. PCRs were carried out using the following parameters: pre-denaturation for 2 min at 96 °C, followed by eight cycles of 96 °C for 15 s, 50 °C for 30 s, and 70 °C for 90 s. DNA concentration of amplicons of interest was determined by gel electrophoresis. About 20 ng amplicon DNA of each sample were pooled and purified with one volume Agencourt AMPure XP beads (Beckman Coulter, Nyon, Switzerland) to remove primer dimers and other small mispriming products, followed by an additional purification using a MinElute PCR Purification Kit (Qiagen, Venlo, the Netherlands). About 100 ng of the pooled amplicon DNA was used to construct a sequencing library using the Ovation Rapid DR Multiplex System 1–96 (NuGEN, San Carlos, CA, USA). The library was size-selected by gel electrophoresis and sequenced from both ends for 300 cycles on the Illumina MiSeq using MiSeq v3 Reagent Kit (Illumina, San Diego, CA, USA) at LGC Genomics (Berlin, Germany). Demultiplexed FASTQ files were generated from base-calls using Illumina’s bcl2fastq v1.8.4 software. Reads with incorrect barcodes, missing barcodes, or conflicting barcode pairs were discarded. A maximum of three mismatches per primer were allowed. After removal of primer sequences using proprietary LGC Genomics software, forward and reverse-complemented reverse reads were merged using BBMerge form the BBMap_34.48 package (http://sourceforge.net/projects/bbmap/) with minimum overlap of 12 bases and a maximum of 3 mismatches.

### Sequence analysis

Sequence filtering was performed using the command trim.seq in MOTHUR v1.35 [15]. The reads that contained ambiguous bases or homopolymer runs longer than 12 bases were removed. Then, sequences were truncated at the beginning of a 20-base window with an average Phred quality <30. Sequences that, after trimming, had a length <300 bases were discarded.

Denoising and clustering of 16S rDNA sequences were made by OTU mapping with the Greengenes reference database [12] pre-clustered at 97 % identity (Greengenes file 97_otus.fasta as of 17 May 2013) using USEARCH (−usearch_global –wordlength 30 –id 0.97 –query_cov 0.9 –top_hits_only) [16]. Sequences with no hits were discarded. For sequences with multiple best hits, the hit that corresponded to the Greengenes reference sequence most frequently assigned in the entire dataset was retained. The reads were classified using naïve Bayesian method and the RDP reference database [13] via MOTHUR (command classify.seqs with options –method = wang and –cutoff = 80) and MOTHUR files trainset10_082014.rdp.fasta and trainset10_082014.rdp.tax. The consensus taxonomy of an OTU was defined as the taxonomy that represented most of the reads within this OTU.

### qPCR

qPCR assay was performed on an Mx3005P qPCR system (Agilent Technologies, Santa Clara, CA, USA). Reaction mixtures contained 12.5 μl of 2× Brilliant II SYBR Green QPCR Master Mix (Agilent Technologies), 0.75 μl of 1/250 diluted reference dye (Agilent Technologies), 0.3 μl of each 25 μM forward (5’-ACTCCTACGGGAGGCAGCAGT-3’) and reverse (5’- ATTACCGCGGCTGCTGGC-3’) primers [17], 1 μl of DNA extract, and 10.15 μl water. The primers used amplify the V3 region of bacterial 16S rRNA genes (*E. coli* positions 338–534). The cycling conditions included initial denaturation of 10 min at 95 °C followed by 40 cycles of 95 °C for 30 s and 68° for 1 min. No-template qPCR controls were performed using 1 μl ddH_2_O instead of DNA extract. The reference curves for DNA quantitation were obtained using known concentrations of genomic DNA of *E. coli* strain DH5α and *S. aureus* strain MW2. All reactions were carried out in duplicate.

### Ethics approval and consent to participate

Not applicable.

### Consent for publication

Not applicable.

### Availability of data and materials

The dataset supporting the conclusions of this article is available in the MG-RAST repository [18] under the project ID 14505 (4647144.3–4647209.3).

## Additional files

Additional file 1: Figure S1. Relative abundance of bacterial genera before and after the decontamination procedure. Genera with mean relative abundance >0.5 % in negative extraction controls are presented. The proportion is indicated by the scale at the bottom of the plot. The R-OTU (ratio between mean ‘absolute’ abundance of OTUs in negative extraction controls and culture samples) cut-off of 0.01 was applied for decontamination. This ratio was calculated from the relative OTU abundance and qPCR data obtained using the *S. aureus* standard curve. For a given culture/dilution or negative extraction control, the data obtained from DNA extractions performed at three different time points (Exp1–Exp3) are presented from left to right. Dilutions of the master stock are indicated from 1E0 (no dilution) to 1E-8 (10^−8^). NEC_W, negative extraction controls obtained by substituting culture for water; NEC_B, negative extraction controls obtained by substituting culture for lysis buffer. (PDF 13 kb) Additional file 2: Figure S2. Effect of *in silico* decontamination on taxonomic profiles of culture dilutions and negative extraction controls. Means for three samples obtained in separate DNA extraction experiments are given. The R-OTU (ratio between mean ‘absolute’ abundance of OTUs in negative extraction controls and culture samples) cut-offs of 1 to 0.001 were applied for decontamination. This ratio was calculated from the relative OTU abundance and qPCR data obtained using the *E. coli* standard curve. Dilutions of the master stock are indicated from 1E0 (no dilution) to 1E-8 (10^−8^). EC, *E. coli*; SA, *S. aureus*. NEC_W, negative extraction controls obtained by substituting culture for water; NEC_B, negative extraction controls obtained by substituting culture for lysis buffer; ND, no decontamination was performed. (PDF 16 kb)

## Abbreviations

NEC: negative extraction control
NEC_B: negative extraction control obtained by substituting culture for lysis buffer
NEC_W: negative extraction control obtained by substituting culture for water
NTC_W: no-template PCR (or qPCR) control
OTU: operational taxonomic unit
R-OTU: the ratio between mean ‘absolute’ abundance of OTUs in NEC and culture samples
