# Supplementary figures and images for: Decontamination of 16S rRNA gene amplicon sequence datasets based on bacterial load assessment by qPCR
Source: BMC Microbiol. 2016 Apr 23;16:73. doi: 10.1186/s12866-016-0689-4 (PMC4842273; doi:10.1186/s12866-016-0689-4)

### Escherichia coli

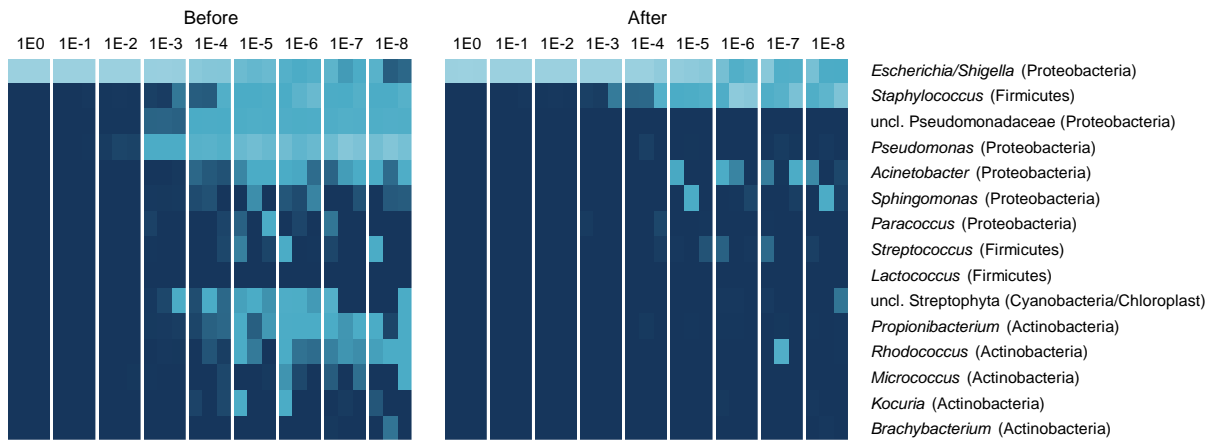

### Staphylococcus aureus

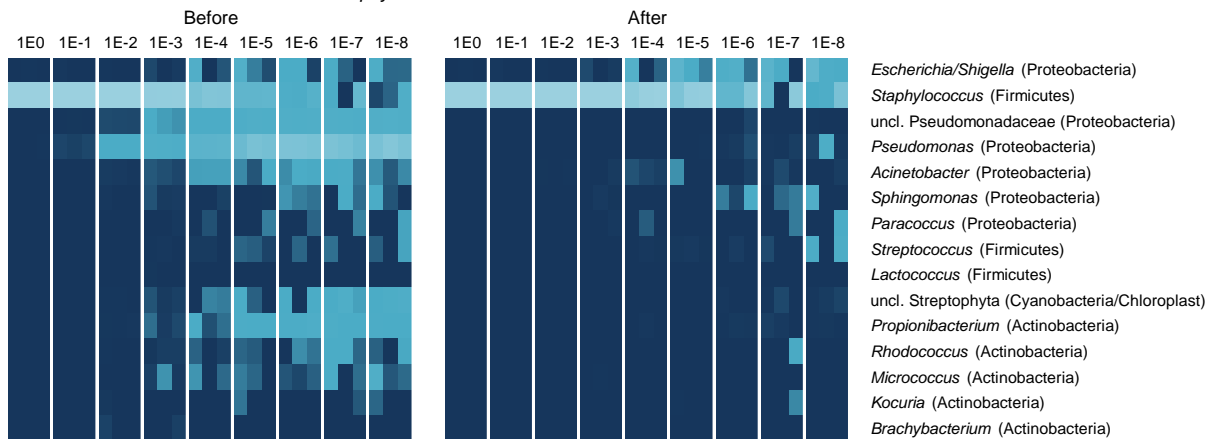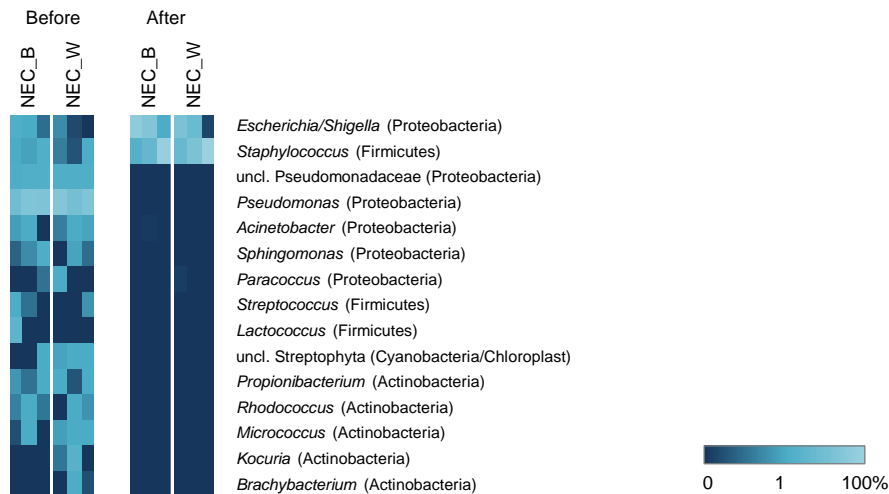

Supplement: Additional file 1: Figure S1. — Relative abundance of bacterial genera before and after the decontamination procedure. Genera with mean relative abundance >0.5 % in negative extraction controls are presented. The proportion is indicated by the scale at the bottom of the plot. The R-OTU (ratio between mean ‘absolute’ abundance of OTUs in negative extraction controls and culture samples) cut-off of 0.01 was applied for decontamination. This ratio was calculated from the relative OTU abundance and qPCR data obtained using the S. aureus standard curve. For a given culture/dilution or negative extraction control, the data obtained from DNA extractions performed at three different time points (Exp1–Exp3) are presented from left to right. Dilutions of the master stock are indicated from 1E0 (no dilution) to 1E-8 (10−8). NEC_W, negative extraction controls obtained by substituting culture for water; NEC_B, negative extraction controls obtained by substituting culture for lysis buffer. (PDF 13 kb) [file 12866_2016_689_MOESM1_ESM.pdf]

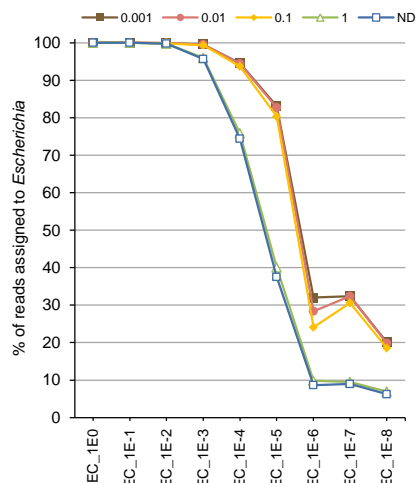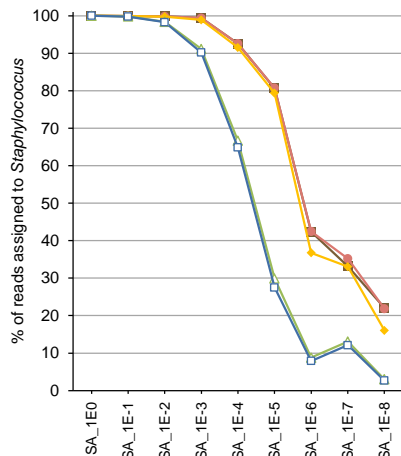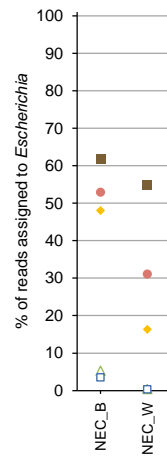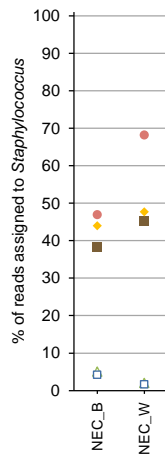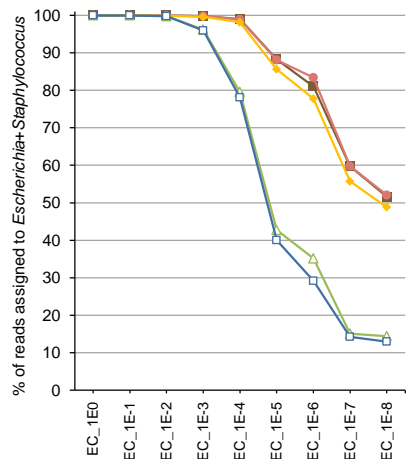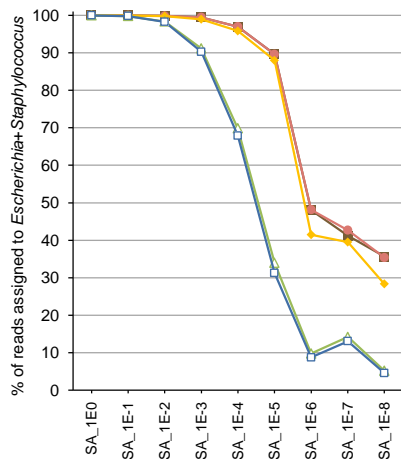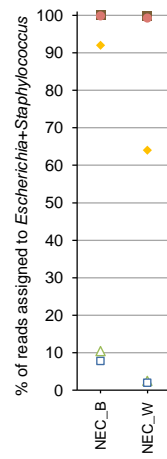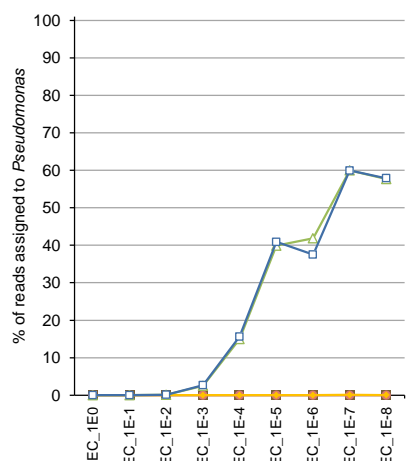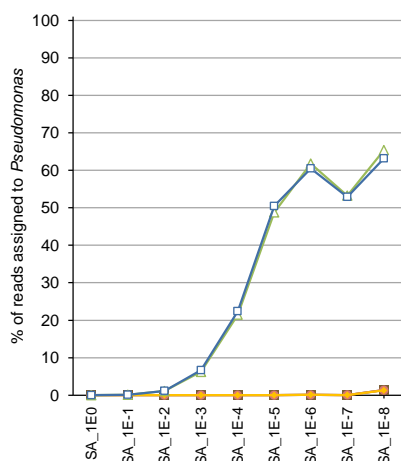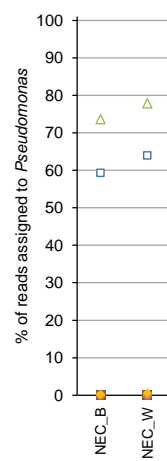

Supplement: Additional file 2: Figure S2. — Effect of in silico decontamination on taxonomic profiles of culture dilutions and negative extraction controls. Means for three samples obtained in separate DNA extraction experiments are given. The R-OTU (ratio between mean ‘absolute’ abundance of OTUs in negative extraction controls and culture samples) cut-offs of 1 to 0.001 were applied for decontamination. This ratio was calculated from the relative OTU abundance and qPCR data obtained using the E. coli standard curve. Dilutions of the master stock are indicated from 1E0 (no dilution) to 1E-8 (10−8). EC, E. coli; SA, S. aureus. NEC_W, negative extraction controls obtained by substituting culture for water; NEC_B, negative extraction controls obtained by substituting culture for lysis buffer; ND, no decontamination was performed. (PDF 16 kb) [file 12866_2016_689_MOESM2_ESM.pdf]
